# An Imitator Within: A Case of Appendiceal Neuroma Masquerading as Chronic Appendicitis

**DOI:** 10.7759/cureus.83986

**Published:** 2025-05-12

**Authors:** Zhen Yun Siew, Ghee Khang Ong, Chia Ting Khoo, Siew Tung Wong, Kenny Voon

**Affiliations:** 1 School of Pharmacy, University of Nottingham Malaysia, Semenyih, MYS; 2 School of Medicine, IMU University, Kuala Lumpur, MYS; 3 School of Biosciences, University of Nottingham Malaysia, Semenyih, MYS; 4 Department of Pathology and Pharmacology, School of Medicine, IMU University, Kuala Lumpur, MYS; 5 Division of Biomedical Sciences, University of Nottingham Malaysia, Semenyih, MYS

**Keywords:** appendicitis, chronic, fibrous obliteration, gastroenteritis, neuroma

## Abstract

The incidence of appendicitis, an inflammation of the appendix, has been rising significantly in many countries worldwide. Appendicitis may present in various forms, including acute or chronic inflammation, and can manifest as either uncomplicated or complicated conditions, etc. Among these conditions, chronic appendicitis is a rare type of appendiceal inflammation that is less common than acute appendicitis. In this case report, we describe a case of idiopathic appendiceal neuroma, which was initially misdiagnosed as acute gastroenteritis. Diagnosis was further supported by comprehensive laboratory testing, computed tomography and ultrasound imaging, as well as immunohistochemical staining. The patient made a full recovery following a laparoscopic appendectomy. This case highlights appendiceal neuroma as an elusive entity with atypical presentation of appendicitis and chronic histological features, emphasising the critical role of histopathology in establishing a definitive diagnosis.

## Introduction

Acute appendicitis (AA) is a common gastrointestinal inflammation regarded as a surgical emergency that necessitates prompt appendectomy. In the past decade, the incidence was reported at 8.7 per 100,000 individuals, with a prevalence of approximately 230 per 100,000 population nationwide. At the regional level, Southeast Asia accounts for 6.4% of the global incidence and prevalence of appendicitis. In contrast, chronic appendicitis is relatively rare, comprising only 10.1% of all appendicitis cases [1]. However, the epidemiology data of appendicitis are scarce in Malaysia.

The incidence of appendicitis is increasing significantly in many countries worldwide [1]. Appendicitis can occur in many forms, including chronic or acute inflammation, uncomplicated or complicated conditions, etc. Among these conditions, typical AA is the most commonly reported condition. In addition to AA, complicated appendicitis, such as perforated appendicitis, was also a focus area of study. In Malaysia, several comprehensive studies investigating AA and complicated appendicitis have been conducted [2,3]. However, chronic appendicitis is a rare type of appendiceal inflammation that is less common than AA and is often idiopathic [4]. Hence, we aimed to report and discuss a rare case of premalignant chronic appendicitis, known as appendiceal neuroma, which was initially misdiagnosed as food poisoning and treated for gastroenteritis.

## Case presentation

In the first quarter of 2024, a male Asian of Chinese ethnicity in his 20s with no significant medical or surgical history due to gastrointestinal disease was admitted to the hospital after five days of serious and worsening chronic watery diarrhoea. The first symptom presented was a low-grade fever approximately 12 hours after he consumed food obtained from a local Ramadan bazaar (festival street food bazaar), followed by intermittent mild abdominal pain localised only to the epigastric and umbilical regions. The fever resolved after the patient took paracetamol and a couple of hours of sleep. Notably, the low-grade fever only presented on the first day. After that, the patient started to experience mild migratory pain and loose diarrhoea. The pain was still limited to the epigastric and umbilical regions only, without progressing to the right lumbar and iliac (right iliac fossa) regions. The watery diarrhoea worsened from having approximately two to three watery stools on the first day to approximately four to five stools per hour on the fifth day (the day of admission). Despite having more than eight hours of sleep per day, the patient complained about fatigue.

During the first diagnosis (four days after abdominal pain presented), the patient had no symptoms other than fatigue, severe watery diarrhoea and migratory abdominal pain. However, his body temperature and blood pressure were normal, and there was no sign of rebound tenderness or tenderness in the right iliac fossa. The patient never complained about nausea, vomiting, anorexia or migratory right iliac fossa pain. The patient suffered from acid reflux on the night of Day 3, but did not experience heartburn. Hence, a tentative diagnosis of food poisoning was assumed by the doctor, and the patient was treated for acute gastroenteritis. Activated charcoal (250 mg tablet), hyoscine butylbromide (10 mg tablet), oral rehydration solutions (4.95 g per sachet) and loperamide hydrochloride (2 mg capsule) were prescribed to the patient. After 24 hours of treatment, the patient’s condition worsened, and he was suffering from extreme migratory abdominal pain when he consumed any food or drinks. Watery diarrhoea persisted.

**Figure 1:**
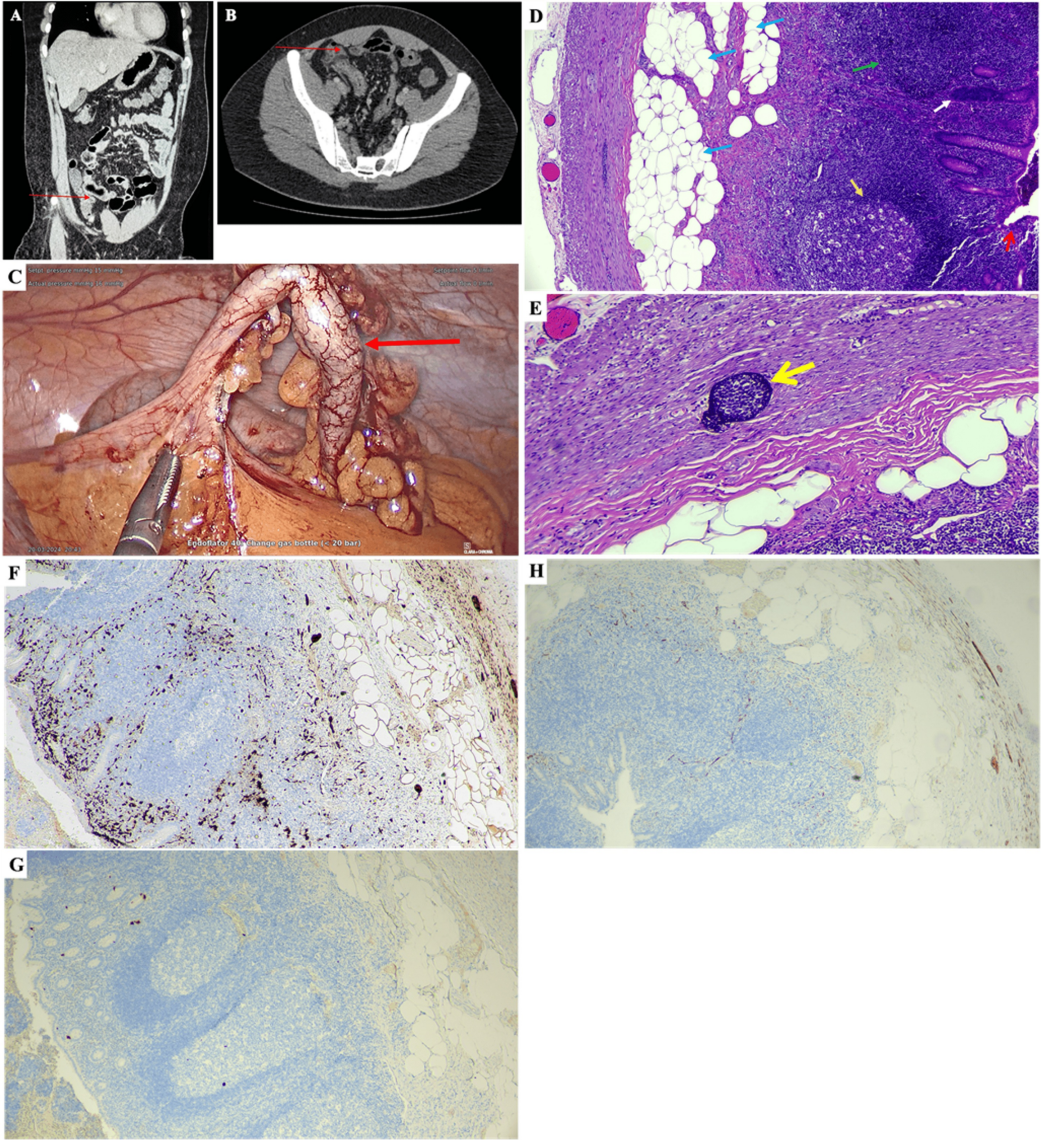
CT-scan images (A, B), a picture of the appendix during surgery (C), micrographs of the biopsied appendix stained with haematoxylin and eosin (D, E), and immunohistochemical staining (F-H). The appendix (red arrow) was identified in the coronal (A) and axial (B) views of the CT-scan image. No focal lesion or mass, no peripancreatic collection, and no streakiness of the adjacent fat were seen. Distended fluid-filled loops of small and large bowel were observed. Thickened distal ileum and caecum were noted. The stomach, small bowel and the rest of the large bowel appeared normal. No obvious bowel-related mass was observed. The appendix was noted to be about 5-6 mm in diameter, and no appendicular mass was observed. (C) Congested blood vessels were observed on the outer surface of the appendix (red arrow), and no perforation can be observed. (D) The cross-sectional view of the appendix (40x magnification) shows fibrosis and fibrofatty tissue infiltration (blue arrows). A reactive lymphoid follicle (gold arrow) was observed surrounded by diffuse lymphoid tissue. Diffuse lymphoid tissue was observed with the loss of the germinal centre (green arrow). Chronic inflammatory cell infiltration (white arrow) was observed in the inner region of a crypt. A mucosal erosion (red open arrow) was noted. Scattered neutrophils were present within the crypt epithelium and mucosa. The wall is infiltrated by mild lymphocytes. Serosa was fibrotic and had no evidence of parasites, granuloma or malignancy. (E) Under 100x magnification, a lymphoid aggregate (yellow open arrow) was observed somewhere in between the submucosa and muscularis layers of the appendix. Immunohistochemical (IHC) staining was performed to stain S-100 protein (F), chromogranin A (G), and synaptophysin (H). Micrographs F-H were captured at 40x magnification.

During the second diagnosis (five days after abdominal pain presented), the patient stopped food intake for at least 12 hours. Despite just drinking plain water, the patient still experienced extreme abdominal pain and had at least four watery stools within an hour. The condition improved greatly when the patient stopped consuming water and food. However, the patient was slightly dehydrated and had slight paleness (pallor). Then, a series of tests and examinations were performed (Table 1 and Figure 1). Based on the tenderness at the patient’s right iliac fossa, CT scan result (Figure 1A-1B), and highly elevated rheumatoid factor and C-reactive protein level, the doctor suggested AA, and a laparoscopic appendectomy was performed on the evening of day five. Intravenous (IV) fluids (Hartmann’s solution and 0.9% NaCl normal saline) were given to the patient for the first 24 hours post-surgery. Ceftriaxone and painkillers were administered for four days (until discharged) and three days (requested by the patient), respectively. After discharge, Zinnat (250 mg tablet, for three days), paracetamol (500 mg tablet) and Pengesic (50 mg capsule) were prescribed to the patient. All symptoms abated after the surgery, and the patient passed normal stool on the second day post-surgery.

**Table 1:** Examinations and tests conducted with results obtained.

Examinations/Tests	Outcomes/Details (On Day 5)
Signs	Tenderness at the right iliac fossa. No rebound pain and fever. Normal blood pressure.
Symptoms	Migratory abdominal pain (from epigastric to umbilical region) stopped after not taking any water or food. No anorexia, nausea, vomiting, acid reflux or heartburn. Fatigue and slight dizziness.
Alvarado score	Scored 6 out of 10. Suggestion: Diagnosis compatible with acute appendicitis, but not convincing enough to warrant appendectomy and were regularly reviewed.
CT scan and ultrasound of the abdomen and pelvis	Axial sections from the dome of the diaphragm till the symphysis pubis. IV contrast was given. The liver and spleen are normal. No focal lesion. The margin is smooth. No dilated intrahepatic duct or common bile duct. The gallbladder is normal, and no stone was seen. The wall is not thickened. The head, neck, body and tail of the pancreas are homogenous. No focal lesion or mass was seen. No peripancreatic collection was noted. No streakiness of the adjacent fat was seen. Distended fluid-filled loops of small and large bowel. Thickened distal ileum and caecum. The stomach, small and the rest of the large bowel appear normal. No obvious bowel-related mass was observed. The appendix is identified as about 5-6mm in diameter, and no appendicular mass was observed. Normal enhancement of both kidneys. No stone or hydronephrosis. Both ureters are identified and of normal appearance, and no ureteric calculi were observed. The abdominal aorta and branches are normal in calibre. No enlarged para-aortic nodes. Normal urinary bladder. The prostate is not enlarged. Subsequent ultrasound shows prominent bowel peristalsis and loops of the bowel. Impression: Features mentioned suggest acute gastroenteritis/enterocolitis changes.
Laboratory tests	
Haematology	Haemoglobin, red cell count (RBC), haematocrit (PCV), MCV, MCH, MCHC, RDW, platelet count, MPV and white blood cell count were normal.
White blood cell differential count	Neutrophil, lymphocyte, eosinophil and basophil counts were normal. Neutrophil to lymphocyte ratio was normal. Monocyte count was elevated: 17.6% (normal = 1 – 11%); 1.19 X 10^3^/μL (normal = 0.20 – 1.00 X 10^3^/μL).
Diabetes mellitus screen	The glucose level was normal.
Renal function and bone metabolism screen	Uric acid, creatinine, urea, sodium, potassium, chloride, calcium and phosphate levels were normal.
Lipid profile	Total cholesterol, triglycerides and non-HDL cholesterol levels, and cholesterol/HDL cholesterol ratio were normal. HDL cholesterol and LDL cholesterol levels were out of range: HDL level was 1.26 mmol/L (normal = >1.42 mmol/L); LDL level was 2.8 mmol/L (normal = <2.6 mmol/L).
Liver function screen	Total protein, albumin, globulin, A/G ratio, total bilirubin, SGOT/AST, SGPT/ALT, alkaline phosphatase, Gamma-GT and alpha-amylase levels were normal.
Blood group	A, rhesus (D) positive.
Thyroid function screen	Free T4 level was normal.
Rheumatoid factor screen	Rheumatoid factor and C-reactive protein (hs-CRP) were both elevated: rheumatoid factor level was 16.3 IU/mL (normal = <15.0 IU/mL); C-reactive protein level was 49.90 mg/L (normal = <5.0 mg/L). C-reactive protein level was about 10 times higher than the normal level.
Viral serology	Dengue IgM, dengue IgG, dengue virus NS1 antigen, total anti-hepatitis A virus and hepatitis B antigen were all non-reactive. Hepatitis B antibody level was 419.8 mIU/mL.
Urine FEME (Urinalysis)	Yellow urine, 1.005 specific gravity, pH 5.0. Negative protein, glucose, bilirubin, urobilinogen and blood in the urine. Ketone was detected in urine.
Microscopic examination - urine	WBC and RBC levels were normal. Epithelial cells were observed occasionally. No cast and crystal were observed.
Macroscopic stool examination	Bristol stool chart: type 6, fluffy pieces with ragged edges. A mushy stool. No blood or mucous was seen.
Typhidot	Typhi IgM and IgG were negative.
Stool culture	Negative for Salmonella typhi, other Salmonella species, Shigella species, Vibrio cholera, Campylobacter species (C. jejuni, C. coli & C. upsiliensis), Clostridium difficile, enteropathogenic Escherichia coli (EIEC, EPEC, ETEC), Shiga-like toxin-producing E. coli (STEC, except EHEC), Yersinia enterocolitica, Cryptosporidium species, Cyclospora cayetanensis, Entamoeba histolytica, Giardia intestinalis, Adenovirus, Astrovirus, Norovirus, Rotavirus and Sapovirus.
Histopathology (biopsy)	Gross description: Specimen of an appendix with mesoappendix measuring 58mm in length and 5mm in widest dimension. The outer surface shows congested blood vessels. The cut surface of the lumen shows a fibrotic wall. The base is painted. Representative section submitted in a single block. Microscopic description: The section of the appendix shows focal obliteration of the lumen at the tip region, replaced by fibrosis and fibrofatty tissue infiltration. Scattered neutrophils are also present within the crypts' epithelium and mucosa. The wall is infiltrated by mild lymphocytes. Serosa is fibrotic. No evidence of parasites, granuloma or malignancy. Diagnosis: Fibrous obliteration with chronic appendicitis.
Immunohistochemistry	Strong S-100 protein stain, weak synaptophysin and chromogranin A stain.

After the surgery, the biopsied appendix was observed with congested blood vessels (similar to Figure 1C). Fibrosis and lipomatosis were observed in the hematoxylin and eosin (H&E) stained specimen (Figure 1D-1E). The immunohistochemical tests indicated a strong S-100 protein staining (Figure 1F), and weak chromogranin A (Figure 1G) and synaptophysin (Figure 1H) staining.

## Discussion

It is believed that appendicitis is caused by obstruction of the lumen, leading to mucous accumulation. The retention of mucus causes appendiceal distension due to an increase in intraluminal pressure and the growth of microorganisms. Abdominal pain located at the right iliac fossa region is caused by the pressure and stimulation on the surrounding sensory neurons, known as the visceral nerve fibres. Eventually, white blood cells, especially neutrophils, will infiltrate into the lumen; inflammation is expected. If the inflamed appendix is not removed soon enough, the necrotic tissue may lead to perforation, which is a form of complicated appendicitis [5]. In contrast, chronic appendicitis, such as fibrous obliteration of the appendix, clinical features may mimic AA, but pathological features are slightly different from AA. Among the pathological features are the loss of the normal appendiceal structure and fibrofatty tissue infiltrations [6]. 

In a review of the literature, a 63-year-old male had undergone a laparoscopic appendectomy, but no localised pain, right-lower abdominal pain, or leukocytosis were observed. He was then diagnosed with fibrous obliteration of the appendix based on the histological examination, despite other laboratory test results (including complete blood cell count and C-reactive protein) being normal. Histologically, focal wall defects and fatty tissue infiltration were observed. Another 43-year-old female diagnosed with fibrous obliteration of the appendix also had no specific abdominal pain during clinical examination [6]. The CT scan images from the two patients mentioned showed some abnormalities, but not in the patient in this case report. It seems like the CT scan and Alvarado score are not always conclusive for chronic appendicitis, and this often causes doctors to misdiagnose chronic appendicitis as other gastrointestinal tract problems.

IHC is not commonly used to diagnose appendicitis. However, in this case report, H&E staining alone is not conclusive enough to confirm the diagnosis. Thus, IHC staining was performed to identify some features of a typical case of fibrous obliteration of the appendix. The rationale for employing specific immunohistochemical markers, S-100, chromogranin A, and synaptophysin, is to elucidate the potential aetiology underlying the presentation of chronic appendicitis. Previous case reports have described both appendiceal neuroma, a premalignant lesion that may precede an appendiceal carcinoid tumour, and the tumour itself as possible clinical manifestations of chronic appendicitis [7-11]. The S-100 protein is typically expressed in appendiceal neuromas, while chromogranin A and synaptophysin are neuroendocrine markers indicative of appendiceal carcinoid tumours. In the present case, strong S-100 protein staining was observed, supporting the diagnosis of appendiceal neuroma [10,11]. The strong S-100 protein staining (Figure 1F) in the appendix confirmed the presence of neurogenic appendicopathy, aligning with histopathological and IHC findings from similar previously reported studies [12-16]. It is important to note that the term "chronic appendicitis" is a broad, non-specific label rather than a definitive diagnosis. It is generally used to describe persistent appendiceal symptoms and may also refer to specific histological features, such as fibrous obliteration of the appendix with chronic lymphocytic infiltration, which is common in appendiceal neuroma [5].

In typical cases of acute appendicitis, patients present within two days of symptom onset, experiencing radiating lower abdominal pain often accompanied by rebound tenderness, guarding, nausea, vomiting, and anorexia [7,8]. In contrast, Mussack *et al*. proposed that lower abdominal pain persisting for more than a week may meet the clinical criteria for chronic appendicitis [17]. Histologically, acute appendicitis is characterised by neutrophilic infiltration into the perivascular regions or muscularis propria of the appendix, along with subserosal vascular congestion [18]. Chronic appendicitis, however, is often defined by fibrous obliteration of the appendiceal lumen with reactive lymphoid follicles and lymphocytic infiltration [7,8,17,19]. Radiographic findings are generally similar between acute and chronic appendicitis [7]. Notably, prior case reports of chronic appendicitis have not included the Alvarado score in their diagnostic approach [7-11]. Unlike those reports, the patient in this case presented with symptoms suggestive of acute appendicitis, yet histopathological analysis revealed features consistent with chronic appendicitis, and immunohistochemical staining confirmed a diagnosis of appendiceal neuroma. The optimal treatment for chronic appendicitis remains laparoscopic appendectomy. Therefore, in atypical cases of appendicitis, relying solely on clinical presentation may lead to misdiagnosis, such as acute gastroenteritis, and should be avoided.

## Conclusions

In conclusion, fibrous obliteration of the appendix remains poorly understood and may be misdiagnosed as gastroenteritis. CT scans and laboratory tests are often inconclusive. Therefore, a laparoscopic appendectomy may be the best approach when symptoms outlined in the Alvarado score are present. In addition to symptoms, modern CT scans and ultrasound studies should guide the decision to proceed with laparoscopic surgery.

## References

[1] Guan L, Liu Z, Pan G, Zhang B, Wu Y, Gan T, Ouyang G (2023). The global, regional, and national burden of appendicitis in 204 countries and territories, 1990-2019: a systematic analysis from the Global Burden of Disease Study 2019. BMC Gastroenterol.

[2] Sukiman H, Mohamad AM, Raduan MF, Yasim MN, Lazim MI (2021). Effect of the Movement Control Order on the Incidence of Complicated Appendicitis During the COVID-19 Pandemic: A Cross-Sectional Study. Malays J Med Sci.

[3] Tan PH, Teng XX, Gan ZY, Tan SQ (2020). A Study on the Clinical Factors Associated with Acute Appendicitis and Perforated Appendicitis among Children in a Secondary Medical Centre in Malaysia. Malays J Med Sci.

[4] Lee CK, Pelenyi SS, Fleites O, Velez V, Alaimo KL, Ramcharan DN, Tiesenga F (2021). Chronic Appendicitis, the Lesser-Known Form of Appendiceal Inflammation: A Case Report. Cureus.

[5] Echevarria S, Rauf F, Hussain N (2023). Typical and Atypical Presentations of Appendicitis and Their Implications for Diagnosis and Treatment: A Literature Review. Cureus.

[6] Choi SJ, Jang YJ, Lee D (2014). Two Cases of Fibrous Obliteration of the Appendix, Mimicking Acute Appendicitis. J Korean Soc Radiol.

[7] Holm N, Rømer MU, Markova E, Buskov LK, Hansen AE, Rose MV (2022). Chronic appendicitis: two case reports. J Med Case Rep.

[8] Almansouri O, Algethmi AM, Qutub M, Khan MA, Mazraani N (2022). A 61-Year-Old Male With Chronic Appendicitis: A Case Report. Cureus.

[9] Gu Y, Wang N, Xu H (2015). Carcinoid tumor of the appendix: A case report. Oncol Lett.

[10] Molina GA, Torres MA, Montenegro MS (2020). Neuroma of the appendix, a rare cause of appendicitis and an important reason for close follow-up. J Surg Case Rep.

[11] Al-Janabi MH, Hasan S, Issa R (2022). Appendiceal neuroma presented as acute appendicitis: A rare case report from Syria. Int J Surg Case Rep.

[12] Dohner E, Kierdorf F, Moreno P, Langer R, Zuber M, Fahrner R (2024). Neurogenic appendicopathy: A rare differential diagnosis of acute appendicitis. J Visc Surg.

[13] Hussein MR, Al Bshabshe A, Elhakeem AA, Elsamman MK (2022). Neurogenic Appendicitis: A Reappraisal of the Clinicopathological Features and Pathogenesis. Diagnostics (Basel).

[14] Partecke LI, Thiele A, Schmidt-Wankel F (2013). Appendicopathy--a clinical and diagnostic dilemma. Int J Colorectal Dis.

[15] Petroianu A, Barroso TV, Buzelin MA, Theobaldo BM, Tafuri LS (2020). Neuroendocrine apendicopathy in morphologically normal appendices of patients with diagnosis of acute appendicitis: Diagnostic study. Ann Med Surg (Lond).

[16] Villar Barroso TV, Petroianu A (2019). Neuroimmunoendocrine peptides on inflammed and morphologically normal appendices removed due to clinical acute appendicitis. Int J Surg.

[17] Mussack T, Schmidbauer S, Nerlich A, Schmidt W, Hallfeldt KK (2002). [Chronic appendicitis as an independent clinical entity]. Chirurg.

[18] Lotfollahzadeh S, Lopez RA, Deppen JG (2025). Appendicitis. [Updated 2024 Feb 12]. In. https://www.ncbi.nlm.nih.gov/books/NBK493193/.

[19] Assefa EA, Shumiye YG, Tesfaye AS, Alemu AK, Ayalew ZS (2024). Chronic appendicitis; the overlooked cause of chronic abdominal pain: Case report. Int J Surg Case Rep.

